# Artificial Intelligence ECG Analysis in Patients with Short QT Syndrome to Predict Life-Threatening Arrhythmic Events

**DOI:** 10.3390/s23218900

**Published:** 2023-11-01

**Authors:** Eros Pasero, Fiorenzo Gaita, Vincenzo Randazzo, Pierre Meynet, Sergio Cannata, Philippe Maury, Carla Giustetto

**Affiliations:** 1 Department of Electronics and Telecommunications, Politecnico di Torino, 10129 Turin, Italy; 2 Cardiology Unit, J Medical, 1015 Turin, Italy; fiorenzo.gaita@gmail.com; 3 Department of Medical Sciences, University of Turin, 10124 Turin, Italy; pierre.meynet@unito.it; 4 Division of Cardiology, Città della Salute e della Scienza Hospital, 10126 Turin, Italy; 5 Department of Cardiology, University Hospital Rangueil, 31400 Toulouse, France; mauryjphil@hotmail.com

**Keywords:** artificial intelligence, shallow learning, deep learning, short QT syndrome, electrocardiogram, sudden cardiac death, risk stratification, vision transformers

## Abstract

Short QT syndrome (SQTS) is an inherited cardiac ion-channel disease related to an increased risk of sudden cardiac death (SCD) in young and otherwise healthy individuals. SCD is often the first clinical presentation in patients with SQTS. However, arrhythmia risk stratification is presently unsatisfactory in asymptomatic patients. In this context, artificial intelligence-based electrocardiogram (ECG) analysis has never been applied to refine risk stratification in patients with SQTS. The purpose of this study was to analyze ECGs from SQTS patients with the aid of different AI algorithms to evaluate their ability to discriminate between subjects with and without documented life-threatening arrhythmic events. The study group included 104 SQTS patients, 37 of whom had a documented major arrhythmic event at presentation and/or during follow-up. Thirteen ECG features were measured independently by three expert cardiologists; then, the dataset was randomly divided into three subsets (training, validation, and testing). Five shallow neural networks were trained, validated, and tested to predict subject-specific class (non-event/event) using different subsets of ECG features. Additionally, several deep learning and machine learning algorithms, such as Vision Transformer, Swin Transformer, MobileNetV3, EfficientNetV2, ConvNextTiny, Capsule Networks, and logistic regression were trained, validated, and tested directly on the scanned ECG images, without any manual feature extraction. Furthermore, a shallow neural network, a 1-D transformer classifier, and a 1-D CNN were trained, validated, and tested on ECG signals extracted from the aforementioned scanned images. Classification metrics were evaluated by means of sensitivity, specificity, positive and negative predictive values, accuracy, and area under the curve. Results prove that artificial intelligence can help clinicians in better stratifying risk of arrhythmia in patients with SQTS. In particular, shallow neural networks’ processing features showed the best performance in identifying patients that will not suffer from a potentially lethal event. This could pave the way for refined ECG-based risk stratification in this group of patients, potentially helping in saving the lives of young and otherwise healthy individuals.

## 1. Introduction

Short QT syndrome (SQTS) is an inherited channelopathy, which was first linked to an increased risk of developing atrial fibrillation [1] and, then, to sudden cardiac death (SCD) [2] in young and otherwise healthy individuals. In 2003, Gaita et al. [2] described two unrelated families with a corrected QT interval (QTc) less than 300 ms and familial history of SCD, outlining SQTS as a novel clinical entity with an autosomal dominant pattern of inheritance. Shortly after, the genetic nature of SQTS was confirmed by the discovery of gain-of-function mutations in potassium channels [3,4,5]. Subsequently, mutations in other channels were described [6,7], even though the yield of genetic screening in these patients remains low (less than 30%).

According to 2022 European Society of Cardiology guidelines [8], SQTS diagnosis is recommended in case of a QTc ≤ 360 ms and one or more of the following: confirmed pathogenic mutation; family history of SQTS; survival from a ventricular fibrillation/tachycardia (VF/VT) episode in the absence of heart disease. Moreover, SQTS diagnosis should be considered in the presence of a QTc ≤ 320 ms or ranging between 320 and 360 ms together with history of arrhythmic syncope; finally, the diagnosis may be considered in case of QTc ranging between 320 and 360 ms and family history of SD below the age of 40 years.

Clinical presentation of SQTS patients is highly heterogeneous; in particular, the most frequent (up to 32%) symptomatic presentation is SCD, which is often the first clinical manifestation of the disease [9]. As a consequence, it is extremely important to discriminate, among asymptomatic patients, those who will experience SCD from those who will not.

Until now, arrhythmia risk stratification in asymptomatic SQTS patients has been suboptimal, since no solid clinical or electrocardiographic parameters predicting life-threatening arrhythmic events are currently available [7,9,10,11,12].

The use of artificial intelligence (AI) in medicine is relatively recent, if compared with other fields (such as speech analytics); however, it is rapidly receiving widespread interest due to high expectations in terms of improving healthcare and reducing related costs [13,14,15,16,17]. In particular, the application of AI in ECG analysis has recently gained tremendous momentum due to the fact that ECG constitutes an ideal substrate for AI application, being a low-cost and widely adopted cardiological tool [18]. Different groups have reported favorable results obtained with AI-based ECG analysis in several clinical settings, such as prediction of underlying atrial fibrillation in patients presenting with sinus rhythm [19], arterial blood pressure estimation [20,21,22,23,24], estimation of age and sex [25], prediction of underlying cardiac contractile dysfunction [26] and of hyperkalemia [27], arrhythmia classification [28,29,30], detection of hypertrophic cardiomyopathy [31], early detection of cardiovascular autonomic neuropathy [32], drug development [33], and, more generally, heartbeat classification [34,35,36]. The high-level discrimination capabilities of such AI models, which have shown very good predictive performances [37,38,39], together with the quickness, availability, and cost-effectiveness of the ECG, highlight the high potential of AI-based ECG analysis. However, to our knowledge, despite its potential, AI-based ECG analysis has never been applied to SCD risk stratification in patients with SQTS.

The purpose of this study was to analyze ECGs from SQTS patients with the aid of different artificial intelligence systems in order to evaluate their ability to discriminate between subjects with and without documented arrhythmic events.

The rest of the paper is organized as follows: Section 2 describes the methodology; Section 3 presents the results, which are then discussed in Section 4; finally, Section 5 yields the conclusions.

## 2. Methods

### 2.1. Definitions and Study Population

The study group included a total of 104 subjects (see Table 1). To our knowledge, this is the first study to use AI for SQTS risk stratification; therefore, to avoid any bias, it was chosen to define SQTS in a very conservative way, as proposed by our group in 2011 [9], as the presence of a QT_c_ interval (Bazett’s formula [40]) ≤ 340 ms. Alternatively, SQTS was defined as a QT_c_ interval ≤ 360 ms (or a QT/QT_p_ ratio ≤ 88%) [9,41] associated with at least one of the following conditions: personal history of SCD, aborted sudden death (aSD) or syncope, or familial history of SCD or SQTS. In total, 84 patients presented with relevant family history: 53 had family history of both SQTS and SCD, while the remaining reported family history only of SCD (*n* = 11) or SQTS (*n* = 20).

A major arrhythmic event was defined as the occurrence of SCD, aborted sudden death (aSD), and/or unexplained syncope. Overall, 37 patients developed a major arrhythmic event, both at presentation and/or during follow-up: 7 died suddenly (SCD), 19 had an aSD, while 11 had unexplained syncope. Conversely, 67 did not experience any major arrhythmic event.

To avoid event-related ECG alterations, the ECGs of patients with major arrhythmic events were sampled far from the event (either before or after). In cases of SCD, since it was not possible to acquire novel ECGs, a baseline ECG was used, recorded before the event occurred. In cases of asymptomatic patients, a baseline ECG was selected from those available.

### 2.2. Dataset Description and Features

For each patient, data regarding both personal and family history were collected together with a 12-lead ECG with a paper speed of either 25 mm/s or 50 mm/s and a gain of 10 mm/mV.

**Figure 1:**
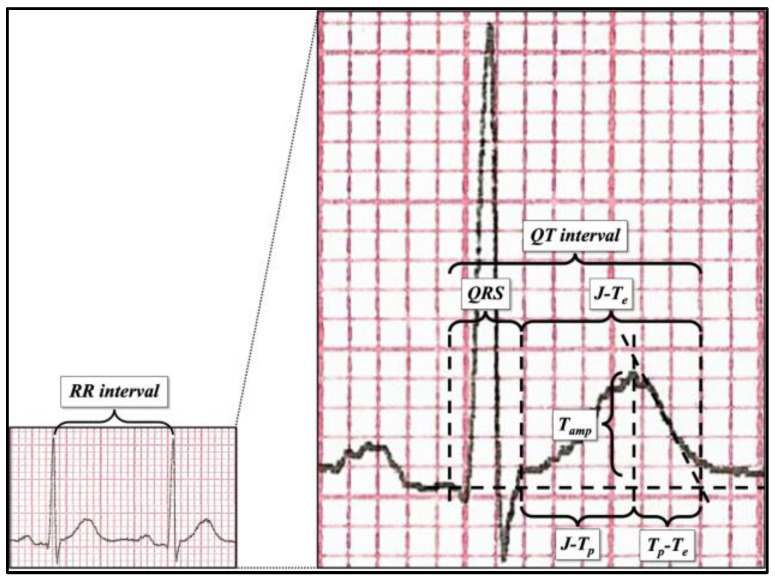
ECG parameter measurement after 400% magnification.

ECG parameters (features) were measured with 400% magnification (see Figure 1) independently by three expert cardiologists, from the lead with the highest T-wave amplitude (usually ranging from V2 to V5), including (see Figure 1 right) RR interval, QT interval, QRS duration, J point–T peak (J-T_p_), T peak–T end (T_p_-T_e_), J point–T end (J-T_e_), and T-wave amplitude (T_amp_). Furthermore, the following parameters were calculated: QT_p_ according to Rautaharju et al.’s formula [41], QT/QT_p_, and the values of QT, J point–T peak, T peak–T end, and J point–T end corrected with Bazett’s formula. QT interval was measured according to the tangential method [40]. The T peak was defined as the highest point of the T wave. The complete feature set is summarized in Table 2.

### 2.3. Neural Networks

Neural networks are a set of algorithms modeled on human brain functions, designed to recognize patterns, i.e., the relationships, between the input and the output (target) signals [42]. In this work, two complementary approaches (human-engineered features vs automatic feature extraction) were tested to perform SQTS risk stratification. In the former scenario, cardiologists measured the features reported in Table 2 and this set was then fed to a shallow learning model, while in the latter case, ECG scans were fed directly to the Vision Transformer without any prior feature extraction phase. Human-engineered features have a direct, clear, medical explanation; for example, the R-R interval refers to the time between two heartbeats. However, the performance of a shallow neural network is highly affected by the input feature choice; since SQTS risk stratification is still an open problem, feature selection is not straightforward. On the other side, Deep Learning models are able to automatically extract the most significant features from the training input images and, therefore, cannot be biased by human-based feature selection. However, they act as a black box, which means they cannot provide any medical explanation of a good risk-stratification performance. It is worth mentioning that, given the limited size of the input dataset, it is probable that a shallow neural network would work better than Deep Learning models.

There are pros and cons in both scenarios; however, given the above, both approaches are complementary and worthy to be explored in an experimental setting.

#### 2.3.1. Shallow Neural Networks: Human-Engineered Features

In a standard multi-layer perceptron (MLP) configuration, the input layer is made of a set of units (neurons), one per each feature, which work as an entry point to the neural network. Indeed, this layer consists of passive nodes, which do not modify the input but only transmit the information to each neuron of the subsequent layer (also known as fully connected). The hidden layer has an arbitrary number of neurons, which depends on the complexity of the problem at hand. Each hidden node combines the information received from each unit of the input layer to achieve a complex representation of the phenomenon under investigation. For this purpose, a non-linear activation function is employed, such as the hyperbolic tangent sigmoid. Finally, the output layer yields the classification of input data by means of the softmax function [43].

Due to the dataset size and the use of human-engineered features, it was chosen to use a shallow learning model. For this purpose, a feed-forward fully connected neural network with one hidden layer was designed. Different hidden-layer sizes were tested to evaluate the corresponding network classification performance; the best performing architecture had 30 and 1 neurons in the hidden and output layers, respectively, while the input layer size depends on the experiment (i.e., on the size of the input feature set). Aside from achieving superior performance, such configuration has a reduced capacity due to the lower number of neurons in the hidden layer. This feature can help prevent overfitting, which is likely to occur when analyzing such small datasets, threatening to invalidate the final results. Hidden units were equipped with hyperbolic tangent sigmoid transfer function, while the output layer used softmax to yield classification. The network training was performed using the scaled conjugate gradient (SCG) [44] technique to minimize the cross-entropy error function.

#### 2.3.2. Shallow Neural Networks: Signals

**Figure 2:**
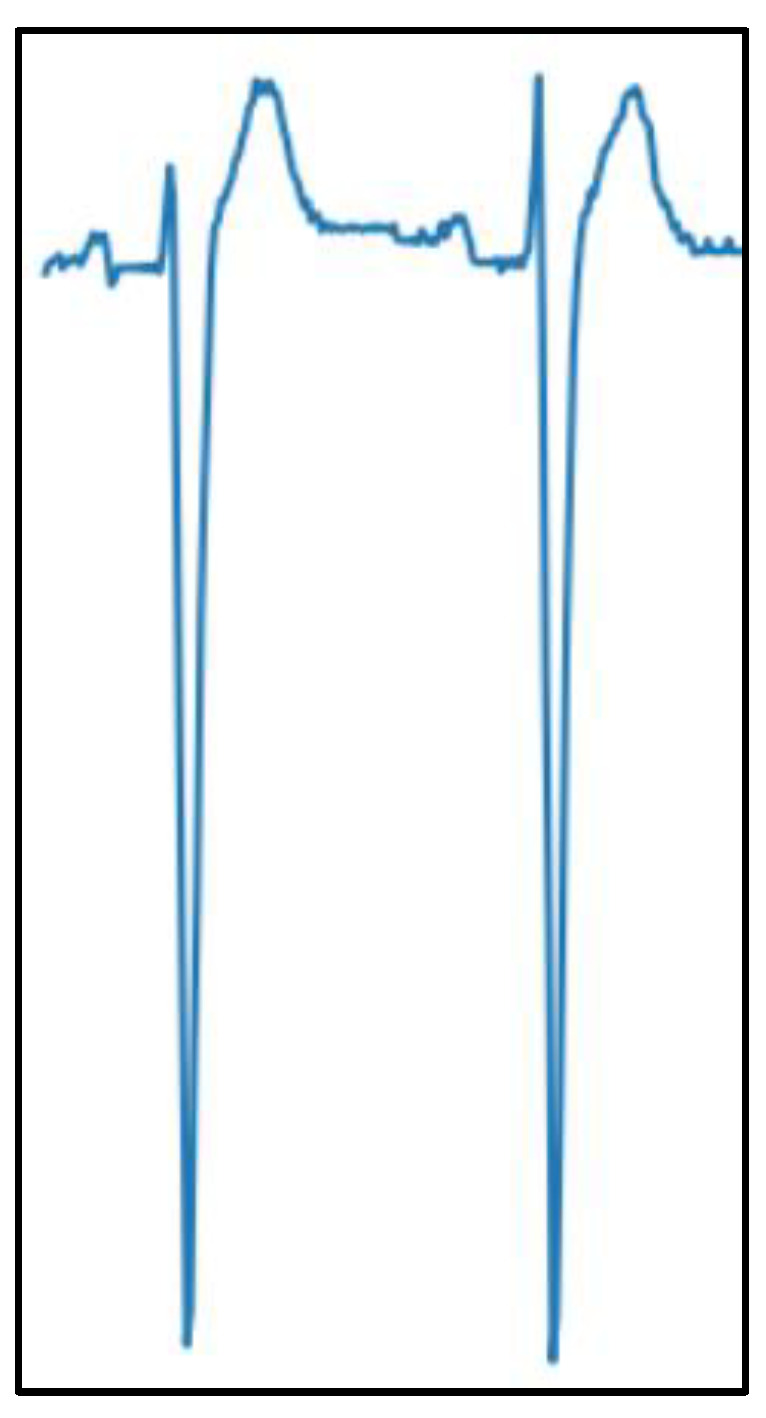
Example of a digitized ECG signal extracted from a scanned image.

A basic multi-layer perceptron was also fed with ECG signals extracted from the two-heartbeat image crops, which are detailed in the next sections. A signal extraction tool [45] was used, yielding 500-sample numerical signals for precordial leads (V1 to V3, since leads V4 to V6 were too noisy to be digitized on several images). This process was remarkably complicated and time-consuming. An example of an extracted numerical ECG signal is shown in Figure 2.

Several configurations for the neural network were tested; the best-performing one had 50 neurons in the hidden layer and 1 neuron in the output layer. The same considerations for the feature approach apply for this case.

#### 2.3.3. Deep Learning Models: Convolutional Neural Networks

Despite being arguably surpassed by more recent models, convolutional neural networks (CNNs) are still the most common deep learning models for computer vision applications. CNNs apply different *kernels* over the input image in order to extract relevant features [46]. Stacking several layers, each one with a different kernel in charge of capturing a specific aspect of the picture, eventually allows the network to collect enough information to execute tasks like classification, segmentation, object detection, and the like.

CNN architectures deployed for this work included EfficientNetV2S, MobileNetV3, and ConvNextTiny [47,48,49], together with a 1-D CNN applied to numerical signals extracted from the images. Apparently, this approach did not yield the expected results, as detailed in the next sections.

#### 2.3.4. Deep Learning Models: Vision Transformer and Swin Transformer

A different type of approach is offered by the Vision Transformer (or ViT), a deep neural network designed as a “computer vision version” of the original Transformer [50,51].

This architecture processes images by dividing them into equally sized patches, which are subsequently embedded and fed to the transformer. The embedding process also accounts for patch position within the image, thus retaining the positional information of each patch. The resulting vector is then processed inside the Transformer encoder by blocks called heads, which exploit the attention mechanism [52] to evaluate the information associated with each patch and how these “patches of information” are related to each other. Multiple heads perform these operations at once, allowing the network to gather knowledge about the global context of the picture. The encoder output is then fed to a multi-layer perceptron to classify the image on the basis of the information that the network was able to extract from it.

The stages of the Vision Transformer in image analysis are in many ways similar to what the human eye and brain do when looking at a picture, trying to grasp its meaning by merging information coming from details and knowledge gathered from the global picture, providing a tool that is able to extract features autonomously. Conversely, shallow learning models require ECG features as inputs to the network, thus implying the necessity to gather medical knowledge about the topic before network deployment.

A different version of the Vision Transformer, called *Shifted Window Transformer* (Swin Transformer) was developed in an attempt to make the basic ViT architecture better suited to vision tasks [53]. In fact, visual entities can undergo large variations, and image pixels can have significant resolution when compared to words in text. In order to overcome these issues, the Swin Transformer’s hierarchical architecture can adapt to different scales, and its computational complexity varies linearly with image size.

This model was applied to ECG images to assess performance against the Vision Transformer. Both Vision and Swin Transformers were pretrained on the ImageNet database [54] and fine-tuned on scanned ECG images. In addition, a 1-D version of the original Transformer encoder was applied to numerical signals extracted from the images.

#### 2.3.5. Deep Learning Models: Capsule Neural Networks

Capsule neural networks (CapsNets) were designed to overcome some of CNNs’ main limitations, like lacking the capability to preserve spatial relationships among image elements [55]. In fact, to mention an “infamous” example, a CNN would typically identify a human face even when its elements—e.g., nose, mouth, eyes—were misplaced with respect to where they were expected to lie. With the introduction of *capsule modules* and *dynamic routing*, these neural models are able to capture the orientation of parts within an image.

#### 2.3.6. Logistic Regression

One of the most common machine learning algorithms, logistic regression fits input data along a sigmoid function, assigning it to different classes according to where itlies along the function plot [56]. This classifier can be fed with images to perform classification, and in this case, it was applied to scanned ECG images.

### 2.4. Data Pre-Processing

The input dataset was pre-processed to enhance network training and avoid overfitting.

In the case of the shallow learning model, to reduce noise in the data and avoid bias in the network training, data were statistically normalized (*z score*) to make the network able to intrinsically determine the importance of each input feature for classification. Indeed, without this step, it would have been possible that some features masked some others, preventing the network from understanding the real contribution of each input attribute to SQTS risk stratification.

**Figure 3:**
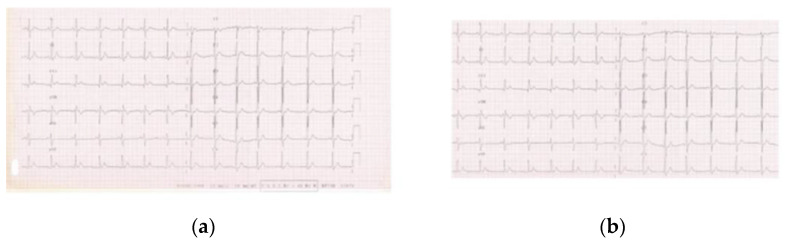
(**a**) ECG image before cropping; (**b**) ECG image after cropping.

On the other hand, for the deep learning models, images were initially cropped to remove all the elements in the bordering part that did not strictly belong to an ECG chart. These elements are usually accompanying information (e.g., annotations) and are considered to carry no relevant data for the given task. Yet, if not removed, their information content could erroneously be marked as noteworthy by the model, thus introducing unwanted biases in classification. Figure 3 shows an example of an ECG image before and after the initial cropping.

**Figure 4:**
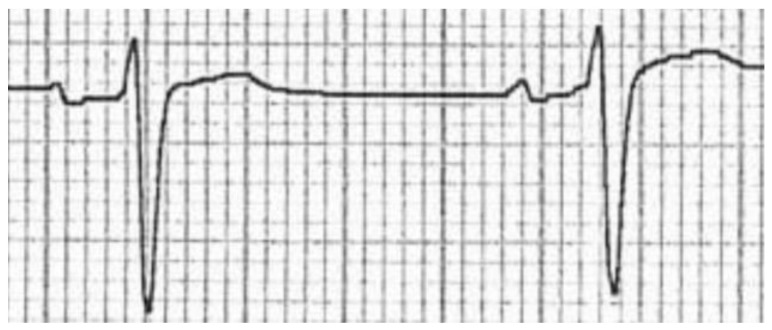
Two-heartbeat crop of a single precordial lead from a scanned image.

In order to try and isolate possible elements of the image carrying more information, images were later cropped to contain only two heartbeats, leading to two additional versions of the dataset: one containing two-heartbeat crops for each precordial lead (e.g.,: V1 only, V2 only, and so on, see Figure 4) and another one containing all two-heartbeat crops for all precordial leads (which are considered to hold more information than peripheral leads with respect to SQTS diagnosis).

Subsequently, data augmentation techniques were implemented for all deep learning models in order to help the network achieve better results during training. In particular, input images went through the following stages, undergoing the RandAugment method for image augmentation [57]: cropping at center; normalizing; horizontal flipping with a 50% probability of rotation occurring; random cropping and resizing; final resizing to the initial size. This process was especially necessary to try to level out the strong data imbalance between the two classes, which would otherwise lead to a penalization of the model’s generalization capabilities.

Another approach was the extraction of numerical signals from the two-heartbeat crops of leads V1, V2, and V3, with the purpose of trying to retrieve the information retained in the original ECG signals that were acquired by the time the data were collected. This was achieved by exploiting the specific tool mentioned previously in Section 2.3.2.

For all AI models, both the input and target sets were randomly divided into three sets as follows: 70% for training, 10% to validate that the network is generalizing and to stop training before overfitting, and the remaining 20% to independently test the network classification performance. To ensure the input data distribution (i.e., the amount of non-event/event cases) was preserved in the three sets, the random division was performed separately for non-event and event subsets.

### 2.5. Classification Metrics

**Figure 5:**
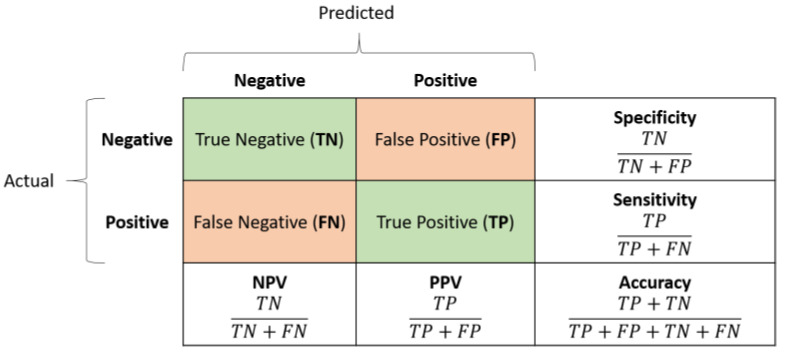
Confusion matrix example: rows yield the real (actual) labels, columns the predicted ones, i.e., the network outputs.

Classification accuracy was estimated by analyzing the confusion matrices and the associated ROC curve. The former, shown in Figure 5, measures the number of times the network correctly classified the input; in this sense, it yields an estimate of how well a single class (negative/positive), i.e., a medical condition (non-event/event), was understood by the neural model. The rows and the columns correspond to the actual (i.e., target) and predicted classes, respectively. The diagonal cells correspond to the true observations (true positive and true negative) correctly classified, while the off-diagonal cells correspond to the false observations (false positive and false negative) incorrectly classified.

To better analyze the network performance, five advanced classification metrics can be derived from the confusion matrix:

- *Sensitivity*, also referred to as *True Positive Rate* or *Recall*, measures the percentage of positive examples correctly labelled as positive by the classifier. In medicine, highly sensitive tests are generally used for screening purposes due to their ability to rule out the disease/event occurrence.
- *Specificity*, also known as *True Negative Rate*, measures the percentage of negative examples correctly labelled as negative by the classifier. In medicine, highly specific tests are typically used for confirmation purposes due to their ability to rule in the disease/event occurrence.
- *Positive predictive value (PPV)*, also known as *Precision*, is the ratio between the total number of correctly classified positive examples and the total number of predicted positive examples. It yields the correctness achieved in positive prediction, which means it measures the likelihood that an event will truly occur given the corresponding network’s positive outcome.
- *Negative predictive value (NPV)* is the ratio between the total number of correctly classified negative examples and the total number of predicted negative examples. It yields the correctness achieved in negative prediction, which means it measures the likelihood that an event will truly not occur given the corresponding network’s negative outcome.
- *Accuracy* refers to the percentage of correct predictions. It is an average measure of the network quality.
- *F1 Score* is the harmonic mean of PPV and sensitivity. It is better suited than accuracy for unbalanced datasets.

Although accuracy provides a single global measure of the classification quality, it is just a mean value of the network performances. In contrast, the area under the ROC curve (AUC) yields a more precise measure (the higher the better) of the predictive accuracy because it represents the probability that a randomly chosen positive sample is ranked higher than a corresponding negative one.

## 3. Results

The classification ability of the proposed shallow learning model was tested on different input configurations, i.e., different input human-engineered feature sets, to study which features were the most relevant to correctly discriminating subjects who will have an event from those who will not. In this sense, the importance of the QT interval and that of the T wave in distinguishing the two classes (i.e., non-event/event) were investigated. Therefore, the experiments could be grouped into five categories as per Table 3:

- QT: only the QT-related features were considered;
- T_wave_: only the T-wave features were considered;
- QT + T_wave_: both the QT-related and T-wave features were considered;
- T_wave ext_: T-wave features were considered together with their Bazett-corrected values;
- All: all input features were considered.

To assess the network classification performances, sensitivity, specificity, PPV, NPV, and accuracy were evaluated. The results for the test set (see Table 4), which checked the ability of the models to perform on new and previously unseen samples, can be summarized as follows:

- Sensitivity: this was generally low in all configurations, with a maximum value of 63.6% in the T_wave_ input configuration and a minimum of 36.4% in the All configuration.
- Specificity: this metric was generally high across all the different explored input configurations, with values ranging from 85% (T_wave_) to 95% (QT, T_wave ext_, and All).
- PPV and NPV: these two metrics did not show optimal values in any of the proposed input configurations; in particular, PPV showed better results compared with NPV (maximum PPV: 83.3% in QT and T_wave ext_; maximum NPV: 81% in T_wave_).
- Accuracy: this evaluation metric was generally suboptimal across all the evaluated configurations, with all the configurations showing 77.4% accuracy with only the exception of the All input configuration, which showed slightly reduced accuracy in the test set (74.2%).
- F1 Score: this evaluation metric was generally suboptimal across all the evaluated configurations, with the T_wave_ configuration reaching the highest value of 66.6 and QT + T_wave_ showing roughly the same performance (63.1).

Finally, Table 5 reports the AUC values for the five evaluated feature sets. While the training-set AUCs are generally satisfying, the same cannot be said for the test-set AUCs. In fact, as can be appreciated, AUC values drop to poor values (AUC < 0.60) or just acceptable values (AUC 0.60–0.70) in all the configurations, except for the QT + T_wave_ configuration, which also presents a good AUC in the testing set (0.81).

Table 6 and Table 7 summarize the results for the other approaches (signal and image analysis). Reported metrics are the test accuracy and the AUC scores obtained by feeding the networks with signals and single lead images. Results are quite similar, regardless of the input type and/or the network architecture: apparently, none of those methods was able to capture any significant difference between the two categories, interpreting every input as a case without an SCD event.

### Comparison with Classical Machine Learning Algorithms

Since the dataset is very small (104 samples and at most 13 features), the one-hidden-layer perceptron is not guaranteed to perform better than other classical ML models. Therefore, to better assess the quality of the proposed shallow learning model, an additional comparison was performed with classical machine learning algorithms including logistic regression [58], decision tree [59], boosted decision tree [59], bagged decision tree [60], support vector machine (SVM) [59], and k-nearest neighbors (KNN) [61]. Also, a PCA feature-selection technique [62] was employed as preprocessing, retaining 90% of the overall explained variance, to see whether reducing the input space would imply an improvement in the classification [63].

Table 8 yields the results for the test set, where different settings of these methods are reported. In more detail:

- Fine tree, medium tree, coarse tree: decision trees with Gini’s diversity index as split criterion and a maximum number of splits equal to 100, 20, 4, for fine, medium, and coarse trees, respectively.
- Boosted decision tree: ensemble of decision trees using the AdaBoost algorithm (maximum number of splits: 20, number of learners: 30, learning rate: 0.1).
- Bagged decision tree: ensemble of decision trees [59] using Bag algorithm (maximum number of splits: 72, number of learners: 30).
- SVMs: support vector machines [59] with different kernel functions, i.e., linear, quadratic, cubic, Gaussian with kernel scale equal to 0.5 (fine Gaussian), 2 (medium Gaussian), and 8 (coarse Gaussian).
- Fine KNN, medium KNN, coarse KNN: k-nearest neighbors algorithm using Euclidean distance as the metric and numbers of neighbors equal to 1, 10, 100, for fine, medium, and coarse KNN, respectively.
- Cosine KNN: k-nearest neighbor algorithm using a number of neighbors equal to 10 and cosine distance as the metric.
- Cubic KNN: k-nearest neighbor algorithm using a number of neighbors equal to 10 and Minkowsky distance as the metric.

Despite some of these methods showing promising results (i.e., 71.0% accuracy), none reached the same accuracy as the shallow learning (i.e., 77.4%). Only the coarse tree with PCA arrived at 74.2% accuracy, still below that of the shallow learning. The results for PCA behavior are not conclusive, since in some cases it improved performances, while in some other cases it worsened them, even for the same technique; as an example, see medium tree vs coarse tree, or quadratic SVM vs cubic SVM.

## 4. Discussion

SQTS is an inherited channelopathy related to increased risk of SCD. SCD is often the first symptomatic presentation, demanding an important effort to better stratify the risk of arrhythmia in patients who are still asymptomatic at medical evaluation. Until now, risk stratification in asymptomatic patients has been unsatisfactory. To our knowledge, this is the first work using an AI-based approach to analyze ECGs in patients with SQTS in order to refine arrhythmia risk stratification. In this study, we used two different AI-based approaches to this purpose: the first approach requires manual feature extraction from the ECG, which is used as input for shallow learning models; other approaches directly use the scanned ECG image or the signal extracted from it as input, automatically performing feature extraction. The main findings are summarized in the following.

Shallow learning models based on different configurations of manually extracted (human-engineered) ECG features achieved suboptimal performance, in particular regarding the NPV, which never exceeded 81%; this is clinically relevant, since it means 2 out of 10 patients with an event were incorrectly classified in the non-event group, potentially leading to under-treatment.

All other approaches did not seem to grasp any significant difference between the two classes and ended up considering each input as a case with no SCD event. There are several possible factors that might have influenced such results:

- Scanned ECG images were extremely different from each other, in terms of resolution, format, color, and background grid color, and most of them suffered from noisy, poor quality; this hindered the possibility of developing a consistent preprocessing procedure that could work efficiently on all dataset images.
- Dataset cardinality was particularly low; this could represent an obstacle for some of the deep learning models chosen, for both the image and the signal approaches. In fact, such architectures often contain a vast number of parameters, and are usually trained on very large datasets.
- Image cropping was performed manually, both for lead isolation and signal digitization; this might have introduced some errors due to the lack of specific methods and criteria for accurate and precise definition of the cropping area.
- The image and signal approaches were conceived to be specular to the feature approach: tested models were supposed to automatically extract relevant features in an unbiased manner, potentially uncovering aspects of the ECG chart that can enrich the knowledge about SQTS and unveiling elements that can hint at an increased risk of an SCD event. Therefore, this methodology cannot leverage any a priori information that could steer the feature search towards a specific direction.

These results suggest that AI-based ECG analysis, in particular using the features approach, might help in refining risk stratification in SQTS patients, supporting clinical decision making in a context where incorrect risk appraisal might translate into the death of young and otherwise healthy individuals. A refined risk stratification means that the clinician may offer the patients the most appropriate treatment to prevent SCD, including cardiac devices. Implantable cardioverter defibrillators (ICDs) still represent the mainstay of treatment for patients with SQTS who are survivors of SCD or have documented spontaneous sustained VT [64], despite significant risk of device-related complications, such as inappropriate shocks (33%), device-related infection (10%), lead failure and fracture (21%), and psychological distress (3.5%) [65,66]. In this sense, better risk stratification might lead not only to earlier adoption of life-saving therapy for patients deemed at higher risk of SCD but also to avoiding implantation of ICDs in low-risk patients, potentially sparing the risk of device-related complications.

### Study Limits

The present work has some limitations, which need to be addressed. First, we acknowledge that the number of patients was limited; however, it should be borne in mind that SQTS is a rare disease, and this constitutes the widest cohort of SQTS patients published so far. Given the limited quantity of ECGSs, it was not possible to refine the analysis into different subgroups, since the results would not have been significant from a statistical point of view.

For the shallow learning model, ECG features were not automatically extracted, but manually calculated by three experienced cardiologists (albeit with 400% magnification to minimize measurement errors), which is prone to errors in manual measurement. Moreover, the study suffers from an implicit bias in terms of the selected features set: although the thirteen features were selected based on the current medical knowledge, they may not be the most relevant to perform SQTS risk stratification. Further studies with different feature sets should be considered. Future works will investigate different methods of feature selection, e.g., L1 regularization, and deepen the relationships among the features and the classification performances.

Finally, to increase generalizability and better assess their reliability, the results presented in this study will be verified in an external SQTS population originating from different regions or medical centers.

## 5. Conclusions

Short QT syndrome is an inherited channelopathy linked with an increased risk of SCD in young and otherwise healthy individuals. Clinical presentation of patients affected by SQTS is highly heterogeneous, with SCD often being the first clinical presentation, and risk stratification is particularly challenging in asymptomatic subjects.

The analysis of ECGs from SQTS patients with the aid of neural networks shows promising results in terms of discriminating between subjects with and without documented arrhythmic events. This could pave the way for refined ECG-based risk stratification in this group of patients, potentially helping in saving the lives of young and otherwise healthy individuals, such as in the initial study performed on Brugada syndrome [59].

Future studies should focus on automatic calculation of the features from digital ECG recordings (either using raw digital ECG data or digitized data from paper-based ECGs). This will guarantee increased reproducibility compared with manual extraction of relevant ECG features. In addition, other deep learning models assessing the whole raw digital ECG signal and/or ECG images should also be explored and should be compared with other architectures. As an example, images will be converted to the frequency domain to apply frequency-domain filters or Wiener filters for noise reduction, so that it will be possible to provide cleaner input to vision-based deep learning models. In parallel, authors will continue to collect SQTS ECGs of subjects who have developed an event, to increase the cardinality of the dataset and further validate the proposed approach; for this purpose, it will probably be necessary to include different cohorts originating from different regions and medical centers. Finally, data augmentation by means of GANs will be explored to increase the quantity of ECG images and, thus, the classification performance.

## Figures and Tables

**Table 1:** Study population characteristics.

Variables	N = 104
Family history, no. (%)	84 (80.8)
SCD	11 (10.6)
SQTS	20 (19.2)
SCD and SQTS	53 (51.0)
Event occurrence, no. (%)	37 (35.6)
SCD	7 (0.7)
aSD	19 (18.3)
Unexplained syncope	11 (10.6)

**Table 2:** Input feature set description.

Feature	Description
**RR (ms)**	Interval between two R waves
**QT (ms)**	Interval from the start of the QRS complex and the end of the T wave (defined using the tangential method); this expresses global duration of ventricular electrical activity, although used to evaluate ventricular repolarization
**QT_c_ (ms)**	QT interval corrected for heart rate using Bazett’s formulaQT_c_ = QT/√RR
**QT_p_ (ms)**	QT interval predicted with Rautaharju et al.’s formulaQT_p_= 656/(1 + HR/100)
**QRS (ms)**	Interval between start and end of the QRS complex; the expresses the duration of ventricular depolarization
**J-T_p_ (ms)**	Interval between J point (junction between the end of the QRS complex and the beginning of the ST segment) and the peak of the T wave; this represents the early phase of repolarization
**T_p_-T_e_ (ms)**	Interval between the peak of the T wave and its end (defined using tangential method); this is a correlate of global dispersion of repolarization
**J-T_e_ (ms)**	Interval between J point (junction between the end of the QRS complex and the beginning of the ST segment) and the end of the T wave (defined using the tangential method); this expresses the effective duration of ventricular repolarization
**T_amp_ (mV)**	Amplitude of T wave measured from the isoelectric line up to its peak
**cJ-T_p_ (ms)**	Interval between J point and the peak of the T wave, corrected with Bazett’s formula
**cT_p_-T_e_ (ms)**	Interval between the peak of the T wave to its end, corrected with Bazett’s formula
**cJ-T_e_ (ms)**	Interval between J point and the end of T wave, corrected with Bazett’s formula
**QT/QT_p_**	Ratio of the QT interval and the QT_p_

**Table 3:** Input dataset taxonomy.

	Input Configurations				
Feature	QT	T_wave_	QT + T_wave_	T_wave ext_	All
**RR (ms)**	✓		✓	✓	✓
**QT (ms)**	✓		✓		✓
**QT_c_ (ms)**	✓		✓		✓
**QT_p_ (ms)**	✓		✓		✓
**T_amp_ (mV)**	✓	✓	✓	✓	✓
**QRS (ms)**	✓		✓		✓
**J-T_p_ (ms)**		✓	✓	✓	✓
**T_p_-T_e_ (ms)**		✓	✓	✓	✓
**J-T_e_ (ms)**		✓	✓	✓	✓
**cJ-T_p_ (ms)**				✓	✓
**cT_p_-T_e_ (ms)**				✓	✓
**cJ-T_e_ (ms)**				✓	✓
**QT/QT_p_**					✓

**Table 4:** Shallow network (human-engineered features) classification performances: sensitivity, specificity, PPV, NPV, accuracy, F1 score. Values are in percentages. The highest values for each metric are highlighted in bold.

	QT	T_wave_	QT + T_wave_	T_wave ext_	All
**Sensitivity**	45.5	**63.6**	54.5	45.5	36.4
**Specificity**	**95.0**	85.0	90.0	**95.0**	**95.0**
**PPV**	**83.3**	70.0	75.0	**83.3**	80.0
**NPV**	76.0	**81.0**	78.3	76.0	73.1
**Accuracy**	**77.4**	**77.4**	**77.4**	**77.4**	74.2
**F1 Score**	58.9	**66.6**	63.1	58.9	50.0

**Table 5:** Shallow network (human-engineered features) classification performances: AUC.

	QT		T_wave_		QT + T_wave_		T_wave ext_		All	
	Training	Test	Training	Test	Training	Test	Training	Test	Training	Test
**AUC**	0.86	0.58	0.75	0.67	0.85	0.81	0.72	0.59	0.76	0.53

**Table 6:** Signal classification approach performances: test accuracy (percentage) and AUC.

	Shallow Network	1-D CNN	1-D Transformer
**Test Accuracy**	63.6	64.0	64.0
**AUC**	0.50	0.51	0.51

**Table 7:** Image classification approach performances: test accuracy (percentage) and AUC.

	EfficientNetV2S	MobileNetV3	ConvNextTiny	Vision Transformer (ViT)	Swin Transformer	Capsule Networks	Logistic Regression
**Test Accuracy**	56.2	56.2	65.6	63.3	64.1	65.0	55.0
**AUC**	0.47	0.47	0.55	0.52	0.50	0.51	0.45

**Table 8:** Accuracy classification performances of state-of-the-art ML methods for the five input configurations, with or without PCA data preprocessing (90% of explained variance retained). Values are in percentages. The highest values in each column are highlighted in bold.

	QT		T_wave_		QT + T_wave_		T_wave ext_		All	
	No PCA	PCA	No PCA	PCA	No PCA	PCA	No PCA	PCA	No PCA	PCA
Logistic Regression	58.1	64.5	64.5	**67.7**	58.1	64.5	58.1	**64.5**	61.3	64.5
Fine Tree	67.7	58.1	48.4	54.8	51.6	**71.0**	54.8	**64.5**	48.4	64.5
Medium Tree	67.7	58.1	48.4	54.8	51.6	**71.0**	54.8	**64.5**	48.4	64.5
Coarse Tree	58.1	**74.2**	58.1	58.1	58.1	67.7	45.2	**64.5**	45.2	58.1
Boosted Trees	54.8	67.7	54.8	64.5	64.5	**71.0**	64.5	45.2	41.9	54.8
Bagged Trees	64.5	67.7	54.8	45.2	**67.7**	64.5	58.1	58.1	58.1	67.7
Linear SVM	64.5	64.5	64.5	64.5	64.5	64.5	64.5	**64.5**	64.5	64.5
Quadratic SVM	**71.0**	58.1	**67.7**	64.5	64.5	**71.0**	54.9	61.3	64.5	**71.0**
Cubic SVM	58.1	64.5	54.8	54.8	58.1	67.7	41.9	58.1	54.8	67.7
Fine Gaussian SVM	64.5	71.0	58.1	58.1	64.5	64.5	61.3	**64.5**	64.5	61.3
Medium Gaussian SVM	64.5	64.5	61.3	61.3	64.5	64.5	64.5	**64.5**	**67.7**	64.5
Coarse Gaussian SVM	64.5	64.5	64.5	64.5	64.5	64.5	64.5	**64.5**	64.5	64.5
Fine KNN	64.5	67.7	64.5	58.1	51.6	58.1	61.3	54.9	51.6	61.3
Medium KNN	54.8	64.5	61.3	61.3	61.3	54.8	**67.7**	58.1	**67.7**	54.8
Coarse KNN	64.5	64.5	64.2	64.5	64.5	64.5	64.5	**64.5**	64.5	64.5
Cosine KNN	54.8	64.5	61.3	61.3	64.5	58.1	**67.7**	58.1	64.5	61.3
Cubic KNN	58.1	64.5	61.3	61.3	61.3	58.1	**67.7**	58.1	**67.7**	58.1

## Data Availability

The data presented in this study are available on request from the corresponding author.
